# Predictors of health-related quality of life in type II diabetic patients in Greece

**DOI:** 10.1186/1471-2458-7-186

**Published:** 2007-07-30

**Authors:** Angelos A Papadopoulos, Nick Kontodimopoulos, Aristidis Frydas, Emmanuel Ikonomakis, Dimitris Niakas

**Affiliations:** 1 Faculty of Social Sciences, Hellenic Open University, Riga Fereou 169 & Tsamadou, 26222 Patras, Greece; 2 Second Department of Internal Medicine, Medical School, Athens University, "Attikon" University General Hospital, Athens, Greece; 3 Plomari Health Center, Lesvos, Greece

## Abstract

**Background:**

Diabetes Mellitus (DM) is a major cause of morbidity and mortality affecting millions of people worldwide, while placing a noteworthy strain on public health funding. The aim of this study was to assess health-related quality of life (HRQOL) of Greek Type II DM patients and to identify significant predictors of the disease in this patient population.

**Methods:**

The sample (N = 229, 52.8% female, 70.0 years mean age) lived in a rural community of Lesvos, an island in the northeast of the Aegean Archipelagos. The generic SF-36 instrument, administered by trainee physicians, was used to measure HRQOL. Scale scores were compared with non-parametric Mann-Whitney and Kruskal-Wallis tests and multivariate stepwise linear regression analyses were used to investigate the effect of sociodemographic and diabetes-related variables on HRQOL.

**Results:**

The most important predictors of impaired HRQOL were female gender, diabetic complications, non-diabetic comorbidity and years with diabetes. Older age, lower education, being unmarried, obesity, hypertension and hyperlipidaemia were also associated with impaired HRQOL in at least one SF-36 subscale. Multivariate regression analyses produced models explaining significant portions of the variance in SF-36 subscales, especially physical functioning (R^2 ^= 42%), and also showed that diabetes-related indicators were more important disease predictors, compared to sociodemographic variables.

**Conclusion:**

The findings could have implications for health promotion in rural medical practice in Greece. In order to preserve a good HRQOL, it is obviously important to prevent diabetes complications and properly manage concomitant chronic diseases. Furthermore, the gender difference is interesting and requires further elucidation. Modifying screening methods and medical interventions or formulating educational programs for the local population appear to be steps in the correct direction.

## Background

Diabetes Mellitus (DM) is a major cause of morbidity and mortality and places a huge strain on public health funding [1]. At present, the disease has reached epidemic proportions, affecting more than 170 million people worldwide, with an estimated increase of at least 50% by 2010, especially in developing countries, and expected to double to about 300 million by the year 2025. Globally, this represents a 42% increase in the number of people with diabetes, producing an approximate overall 27% increase in the prevalence of the disease [2]. Diabetes complications have important effects on patients' quality of life as well as socio-economic implications [3].

Measuring health-related quality of life (HRQOL) in Type II DM is important for several reasons such as dietary restrictions, medication and the actual symptoms of this disease as well as concomitant diseases, all of which may lead to deteriorations in HRQOL. Moreover, the guidelines for treatment of Type II DM emphasize that one of the primary objectives is to improve HRQOL [4]. This implies that HRQOL is increasingly used as an outcome measure to monitor the burden of DM on the population and the results of previous studies show that HRQOL is associated with duration of diabetes [5], age [6], female gender [7,8], diabetic complications [9,10], concomitant diseases [11,12] and disease severity [13].

Many studies of HRQOL in diabetic patients have been performed and comprehensive reviews are available in the literature [14,15]. Various domains of functioning and well-being contribute uniquely to overall HRQOL, implying that a multidimensional measurement approach is required [16]. In light of this, the majority of quality of life studies involving diabetics have been performed using multidimensional assessment including physical, psychological, and social functioning and well-being. The two major approaches to measuring HRQOL are generic and disease-specific instruments, and the two have been compared [17,18] in diabetes patients and shown to demonstrate complementarity and provide different kinds of information, with the generic ones perhaps providing more information than their disease-specific counterparts [19].

The prevalence of diabetes is highest in older adults whose quality of life is of great concern, and many studies have examined the relationship between diabetes and HRQOL among this population [20-22]. Furthermore, the increasing prevalence of diabetes, both in urban [23,24] and rural [25,26] areas in Greece, has been shown. However, there is only a small amount of information on the association of sociodemographic and disease-specific factors with the HRQOL of Greek Type II DM patients. Therefore, the aim of this study was to use a generic multidimensional instrument (SF-36) to identify significant disease-specific and sociodemographic predictors of diabetes in this rural patient population.

## Methods

### Sample and data collection

The subjects in this study were Type II DM patients living in the area served by the Health Center of Plomari, located on the island of Lesvos in the northeastern part of the Aegean Archipelagos. Patients were recruited from the health center's database of 5.986 adults residing within in its service area, and of whom 469 (7.8%) have been identified as diabetics. Trainee physicians met with patients consecutively during their routine visits to the health center or to one of its affiliated rural posts and collected information via interview, clinical observation and by reviewing the patients' medical records, as it has been suggested that these data sources supplement each other in providing reliable clinical data [27]. The study was conducted in the second half of 2005 and 229 patients, out of 254 visiting the health center during the study period, agreed to participate (90.2% response rate). The survey included the SF-36 quality of life instrument, socio-demographic and diabetes-specific questions. The heath center's administration, in cooperation with the review board of the supervising general hospital of Mytilini, granted ethical approval for this study and all subjects provided informed consent.

### Measurement of HRQOL

The SF-36 health survey contains 36 questions covering functional health status and general health, currently and over the previous 4 weeks [28], and has been validated in a Greek general population [29]. The questions are summarized into eight scales measuring physical functioning (PF), role physical (RP), bodily pain (BP), general health perception (GH), vitality (VT), social functioning (SF), role emotional (RE), and mental health (MH), with higher scores (0–100 range) reflecting better-perceived health. Previous studies have shown that the instrument discriminates well between perceptions of people with or without one or more chronic diseases [30-32], and between people with and without diabetes [33-35].

### Statistical Analyses

The SF-36 subscales were scored according to documented procedures [36]. Internal consistency reliability of each scale was calculated using Cronbach's alpha and the 0.70 standard for group-level comparisons was adopted [37]. Normality was tested with the *Kolmogorov-Smirnov *test. Subscale scores were compared within groups, using *Mann-Whitney *and *Kruskal-Wallis *nonparametric tests, for each sociodemographic and diabetes-related independent variable. Multivariate stepwise linear regression analyses (with the eight SF-36 subscales as the dependent variables) were performed to investigate the relationships between HRQOL scores, sociodemographic variables and data concerning the disease and its therapy. Specifically, independent variables included in the analyses were gender, age, marital status, education, employment, BMI, micro- and macrovascular complications, hypertension, hyperlipidaemia, years with diabetes and concomitant chronic diseases. Relationships were considered statistically significant when *p*-values ≤ 0.05 were reached. All analyses were performed with SPSS version 13.0 (SPSS Inc. Chicago, IL, USA).

## Results

Socio-demographic and diabetes-related data are presented in Table 1. Most respondents are female (52.8%) and the mean age, for the entire sample, is 70.0 years. The majority is of low educational status, having completed only primary school (82.1%). Most patients are married (74.0%) and retired (42.7%). Regarding diabetes, the average duration is approximately 10 years. The majority of patients (95.2%) reported suffering from at least one other chronic (non-diabetic) medical condition such as hypertension (76.9%) and/or hyperlipidaemia (42.5%). Diabetic complications are prevalent in this sample. Specifically, 23.6% suffer from microvascular diseases -mostly angiopathy and retinopathy- and 31.4% from macrovascular complications, mostly cardiovascular disease. Finally, most patients control their diabetes via diet (74.9%) and antidiabetic medication (70.9%) and 87.3% regularly take medication (mostly antihypertensive and antilipidaemic) for other health problems.

**Table 1:** Characteristics of the sample (N = 229) of diabetic patients in a Greek rural society

**Demographics**	**N (% valid)**	**Diabetes-related data**	**N (% valid)**
Gender (female)	121 (52.8)	Body mass index (Kg/m^2^) (mean ± SD)	30.4 ± 5.2
Age (mean ± SD)	70.0 ± 9.9	Prevalence of diabetic complications	
Education (years)		**Microvascular**	54 (23.6)
≤ 6	183 (82.1)	Angiopathy	35 (15.8)
7–12	31 (13.9)	Retinopathy	25 (11.1)
> 12	9 (4.0)	Neuropathy	19 (8.5)
Family status		Nephropathy	7 (3.1)
Single	9 (4.0)	**Macrovascular**	72 (31.4)
Married	165 (74.0)	Comorbidity*	218 (95.2)
Divorced/Separated	1 (0.4)	Hypertension	176 (76.9)
Widowed	48 (21.6)	Hyperlipidemia	93 (42.5)
Occupational status		Years with diabetes (mean ± SD)	10.0 ± 8.3
Retired	93 (42.7)	Smoking	21 (9.6)
Farming	46 (21.1)	Diabetes control method	
Other	22 (10.1)	Diet	146 (74.9)
Keeping house	57 (26.1)	Medication	161 (70.9)
		Insulin	27 (11.9)
		Other medication	200 (87.3)

* One or more chronic medical conditions

Central tendency, variability and reliability of the SF-36 scales are presented in Table 2. The percentage of valid responses was high in all scales as a result of interviewing the patients. Two scales, RP and RE, suffer floor effects because the relevant questions are dichotomous (the only ones in the instrument) and generate fewer possible response levels. Concerning reliability, all scales meet the recommended >0.70 internal consistency criterion. The eight subscale scores range from 48.9 for GH to 74.8 for SF. Scores were computed and compared according to sociodemographic characteristics of the sample, and the results are shown in Table 3.

**Table 2:** Central tendency, variability and reliability of the SF-36 subscales

	**No. of Items**	**% N Valid**	**Mean (SD)**	**95% CI**	**Median**	**Floor (%)**	**Ceiling (%)**	**Reliability**^1^
Scales								
								
Physical functioning	10	99.6	64.5 (29.5)	60.7–68.4	75.0	0.4	9.2	0.94
Role physical	4	99.6	62.0 (45.8)	56.0–67.9	100.0	30.7	55.7	0.96
Bodily pain	2	99.6	73.0 (30.5)	69.0–76.9	84.0	2.6	46.1	0.92
General health	5	99.1	48.9 (23.0)	45.9–51.9	47.0	1.8	0.4	0.76
Vitality	4	99.6	56.9 (27.4)	53.0–60.2	60.0	2.6	4.4	0.88
Social functioning	2	98.7	74.8 (29.7)	70.9–78.7	87.5	2.2	44.2	0.84
Role emotional	3	99.6	63.6 (45.0)	57.7–69.5	100.0	29.8	56.6	0.93
Mental health	5	98.7	60.1 (26.3)	56.7–63.5	64.0	0.9	1.3	0.85

^1^Measured with Cronbach's alpha coefficient

**Table 3:** SF-36 subscale scores according to demographic characteristics of the sample (N = 229)

		**PF**	**RP**	**BP**	**GH**	**VT**	**SF**	**RE**	**MH**
		
**Sex**									
Male		74.6	69.2	81.7	53.3	63.3	85.0	72.6	65.7
Female		55.4	55.4	65.3	45.0	50.6	65.8	55.7	55.1
	***P (sig.)***	0.000***	0.016*	0.000***	0.007**	0.000***	0.000***	0.007**	0.003**
**Age**									
≤ 65		74.7	66.7	72.4	51.2	62.4	76.7	65.7	61.4
66–75		62.4	63.8	72.5	47.4	54.4	74.3	64.8	59.3
> 75		56.5	54.5	74.2	48.4	53.3	73.5	59.7	59.8

	***P (sig.)***	0.000***	*0.380*	*0.925*	*0.578*	*0.084*	*0.812*	*0.659*	*0.799*
**Marital Status**									
Married		66.3	64.9	76.2	49.7	59.3	76.4	66.5	62.1
Single/Widowed		59.6	53.5	65.5	46.1	48.4	71.5	55.2	53.6

	***P (sig.)***	*0.078*	*0.078*	0.021*	*0.304*	0.011*	*0.321*	*0.080*	0.031*
Education									
≤ 6 years		60.5	57.3	69.4	46.8	54.0	72.0	60.3	57.8
7–12 years		81.6	81.5	85.6	54.6	65.3	85.9	71.0	65.1
> 12 years		81.7	72.2	91.1	58.9	63.8	80.6	81.5	76.4

	***P (sig.)***	0.000***	0.014*	0.002**	*0.107*	0.049*	0.022*	*0.267*	0.032*
**Work Status**									
Retired		62.0	68.3	78.5	49.9	57.0	79.1	69.9	62.2
Employed/Farming		72.4	57.4	77.7	50.7	60.0	78.2	59.8	61.8
Keeping House		58.6	56.3	61.9	47.3	52.4	64.5	58.5	54.8

	***P (sig.)***	0.013*	*0.159*	0.004**	*0.788*	*0.375*	0.031*	*0.180*	*0.226*

*** *P *<*0.001*, ** *P *<*0.01*, * *P *<*0.05 *according to Mann-Whitney or Kruskal-Wallis test.

Abbreviations: PF = Physical Functioning, RP = Role Physical, BP = Bodily Pain, GH = General Health, VT = Vitality, SF = Social Functioning, RE = Role Emotional, MH = Mental Health

Kolmogorov-Smirnov normality tests showed non-normal distributions for PF, RP, BP, SF and RE (*P *< 0.001), MH (*P *< 0.01) and VT (*P *< 0.05), therefore non-parametric Mann-Whitney and Kruskal-Wallis tests were used for comparisons. Females reported statistically significantly lower quality of life in all domains, specifically PF, BP, VT, and SF (*P *< 0.001), GH, RE and MH (*P *< 0.01) and RP (*P *< 0.05). Regarding age, only PF scores deteriorate significantly in older age-groups (*P *< 0.001), however the scores for the other subscales are in the expected directions as well. This particular sample is comprised mostly of elderly patients, implying that age is not expected to discriminate well between quality of life levels. The same holds for two other social variables, i.e. marital status and education. As 75% of the sample is married and over 80% are of the same (low) educational status, these variables will also be poor discriminators. In any case, married patients reported better health in all scales and the differences were significant for BP, VT and MH (*P *< 0.05). Similarly education is a significant discriminating variable for PF (*P *< 0.001), BP (*P *< 0.01), RP, VT, SF and MH (*P *< 0.05).

Similar analyses were performed using diabetes-related data as discriminators of HRQOL (Table 4). Duration of the disease appears to be the most influential factor as it negatively and statistically significantly affects all SF-36 subscales, especially in the case of the ">10 years" group. Micro- and macrovascular complications also demonstrate a negative influence on six and five quality of life subscales respectively. As expected, the absence of diabetes complications results in improved HRQOL, however statistical insignificance is observed for RP and BP, and for RE in the case of macro-complications. The coexistence of non-diabetic comorbidity generally results in lower scale scores, and the differences are significant for PF (*P *< 0.01), BP, GH and SF (*P *< 0.05). Hypertension and hyperlipidaemia also affect HRQOL in a negative manner, with the most significant differences appearing in the GH and MH subscale scores respectively (*P *< 0.001). Concerning body mass index, patients in the "normal" range (<25 Kg/m^2^) report higher scores than those in the "overweight" and "obese" ranges, however the differences are statistically significant only for PF (*P *< 0.05).

**Table 4:** SF-36 subscale scores according to health-related characteristicsof the sample (N = 229)

		**PF**	**RP**	**BP**	**GH**	**VT**	**SF**	**RE**	**MH**
		
**Body mass index**									
< 25 Kg/m^2^		74.7	74.2	82.7	54.9	66.3	81.5	71.3	69.5
25–30 Kg/m^2^		67.0	58.6	72.8	48.4	54.4	73.6	59.3	58.2
> 30 Kg/m^2^		60.5	62.6	71.7	49.0	56.5	74.7	66.4	59.6
	***P (sig.)***	0.038*	*0.250*	*0.217*	*0.425*	*0.123*	*0.450*	*0.312*	*0.103*
**Microvascular Complications**									
Yes		49.0	50.5	66.6	39.2	46.5	64.2	48.2	51.8
No		69.2	65.4	74.9	51.9	59.7	78.1	68.4	62.7

	***P (sig.)***	0.000***	*0.059*	*0.072*	0.000***	0.000***	0.002**	0.009**	0.009**
**Macrovascular Complications**									
Yes		50.4	52.5	72.1	41.4	46.8	68.8	56.9	54.5
No		70.9	66.2	73.4	52.4	61.1	77.7	66.7	62.7

	***P (sig.)***	0.000***	*0.059*	*0.995*	0.000***	0.000***	0.021*	*0.092*	0.031*
**Hypertension**									
Yes		62.7	60.6	71.8	46.1	54.4	73.1	62.7	58.6
No		70.5	66.5	76.9	58.0	63.8	80.5	66.7	64.8

	***P (sig.)***	0.047*	*0.446*	*0.224*	0.000***	0.027*	*0.118*	*0.527*	*0.126*
**Hyperlipidaemia**									
Yes		58.8	57.9	66.7	45.4	50.4	70.4	58.1	52.7
No		67.1	62.0	76.5	51.0	59.7	77.4	66.4	64.4

	***P (sig.)***	0.049*	*0.287*	0.022*	0.037*	0.016*	*0.079*	*0.176*	0.000***
**Years with diabetes**									
< 5		67.6	59.8	75.7	54.3	61.0	78.9	69.8	65.9
5–10		69.7	74.0	78.7	51.6	61.6	81.1	68.8	61.7
> 10		56.4	52.0	64.6	41.3	47.4	64.8	52.4	53.1

	***P (sig.)***	0.016*	0.017*	0.011*	0.000***	0.002**	0.000***	0.038**	0.008**
**≥ 1 other diseases**									
Yes		61.6	61.6	70.4	46.6	54.5	71.9	62.0	58.8
No		73.8	66.0	79.4	55.4	62.5	83.3	70.4	63.9

	***P (sig.)***	0.004**	*0.595*	0.027*	0.012*	*0.060*	0.022*	*0.208*	*0.188*

*** *P *<*0.001*, ** *P *<*0.01*, *P <*0.05 *according to Mann-Whitney or Kruskal-Wallis test.

Abbreviations: PF = Physical Functioning, RP = Role Physical, BP = Bodily Pain, GH = General Health, VT = Vitality, SF = Social Functioning, RE = Role Emotional, MH = Mental Health

Multivariate analyses for the SF-36 (Table 5) showed that sex (female) had the most pronounced negative influence on HRQOL. Other sociodemographic factors were significant predictors for certain SF-36 subscale scores, e.g. older age is associated with lower PF and RE scores (*P *< 0.01), being married with higher VT (*P *< 0.05), higher education with less BP (*P *< 0.05) and being employed with worse GH (*P *< 0.05) and more emotional role limitations (*P *< 0.01). As for the diabetes-specific factors, microvascular complications and disease duration were the most significant predictors of HRQOL, each affecting negatively and significantly five SF-36 subscales. The presence of other chronic diseases is also a significant predicting factor for four subscales. It appears that PF is the HRQOL dimension most significantly influenced by sociodemographic and disease-related factors, and this is reflected by the high portion of variance explained by the linear regression model (42%). For the other subscales, the models explained portions of variance ranging between 15–26%, except for the RE subscale which appears to be unaffected (R^2 ^= 0.043).

**Table 5:** Multivariate analyses for SF-36 subscales

	**B Coefficient *(p-value)***							
	
	**PF**	**RP**	**BP**	**GH**	**VT**	**SF**	**RE**	**MH**
Constant	147.4 (*P *<*0.001*)	90.0 (*P *<*0.001*)	93.4 (*P *<*0.001*)	26.0 (P = 0.045)	60.2 (*P *<*0.001*)	79.0 (*P *<*0.001*)	164.8 (*P *<*0.001*)	39.5 (*P *= *0.001*)
Female	-18.4 (*P *<*0.001*)	-19.0 (*P *= *0.006*)	-15.0 (*P *= *0.001*)	-8.3 (*P *= * 0.012*)	-12.8 (*P *= *0.001*)	-21.2 (*P *<*0.001*)	-26.2 (*P *<*0.001*)	-11.9 (*P *= *0.002*)
Age (per year)	-1.0 (*P *<*0.001*)						-0.9 (*P *= *0.016*)	
Married					10.0 (*P *= *0.028*)			
Higher education			8.7 (*P *= *0.043*)					
Employed				-8.1 (*P *= *0.029*)			-22.1 (*P *= *0.004*)	
Body mass index (Kgr/m^2^)	-1.5 (*P *<*0.001*)							
Microvascular complications	-14.6 (*P *= *0.001*)			-14.2 (*P *<*0.001*)		-11.7 (*P *= *0.016*)	-19.2 (*P *= *0.015*)	-12.9 (*P *= *0.006*))
Macrovascular complications	-13.3 (*P *<*0.001*)				-12.1 (*P *= *0.004*))			
Hypertension				-9.5 (*P *= *0.010*)				
Hyperlipidemia								-9.0 (*P *= *0.021*)
Years with diabetes (per year)			-0.7 (*P *= *0.007*)	-0.5 (*P *= *0.025*)	-0.6 (*P *= *0.014*)	-0.7 (*P *= *0.006*)		
≥ 1 other diseases	-10.3 (*P *= *0.013*)			-10.3 (*P *= *0.005*)	-10.2 (*P *= *0.022*)	-11.0 (*P *= *0.015*)		

**R^2 ^= **	**0.42**	**0.04**	**0.15**	**0.22**	**0.20**	**0.26**	**0.18**	**0.13**

Abbreviations: PF = Physical Functioning, RP = Role Physical, BP = Bodily Pain, GH = General Health, VT = Vitality, SF = Social Functioning, RE = Role Emotional, MH = Mental Health

## Discussion

We investigated the association of demographic, social and diabetes-specific variables on the HRQOL of a sample of elderly Greek Type II DM patients living on a remote located island in the Aegean archipelagos. Evidence was provided to support assertions made in previous studies, that impaired HRQOL is associated with obesity [38], micro- and macrovascular complications [39], hypertension [40], hyperlipidaemia [41] and non-diabetic comorbidity [42]. Furthermore, age, gender, marital status and education were also confirmed as important discriminators of HRQOL in type II diabetics. All comparisons showed differences in the expected directions and mostly statistically significant. The most influenced SF-36 subscale was PF since the differences, for all the variables studied, were statistically significant except for marital status, which was marginally insignificant (*P *= 0.078). Contrarily, the role limitations scales (RP and RE) were the least affected, most likely due to their poor discriminating ability mentioned previously.

The multivariate regression analyses (Table 5) indicate that diabetes-related variables are more important predictors of HRQOL, compared to demographic and social characteristics of the sample. An obvious exception is gender (female), which appears to be significant overall. Specifically, microvascular complications (mostly angiopathy and retinopathy), disease duration and non-diabetic comorbidity were the most profound predictors of a negative HRQOL, and when combined seemed to affect all SF-36 subscales except for RP, which remained unaffected with only 4.3% of its total variance explained in this study. On the other hand PF, as expected according to the results in tables 3 and 4, is the domain best predicted by this set of variables (R^2 ^= 0.42). Because of the relationships observed in this study, as well as in others, it is reasonable to conclude that any efforts to avoid or postpone obesity, development of complications, hypertension, hyperlipidaemia and other non-diabetic comorbid conditions will enhance HRQOL and thereby improve healthy life expectancy. However, efforts to prevent complications of diabetes often overlook the impact of the condition and its treatment on current quality of life HRQOL, implying potential space for concurrently measuring treatment satisfaction [43].

It is generally accepted that the prevalence of diabetes varies among populations as a result of different environmental influences and genetic susceptibility [44]. Furthermore, it has been clearly shown that urbanization affects prevalence, with higher rates in urban areas than in rural communities [45]. A recent Greek study involving subjects in both urban and rural areas reported an age- and sex-adjusted prevalence of DM of 10.6%, while undiagnosed diabetes was detected in 34% of the cases [46]. In another study involving a municipality of rural Crete, a relatively high age-standardized prevalence of DM of 5.2% was found [25].

In the present study, despite the inability to standardize due to the lack of age and gender information of the entire service population, the prevalence of type II DM in this rural community appears to be relatively high (7.8%). This implies that the findings from this study could have important implications for health promotion in rural medical practice in Greece. In terms of primary care for example, modification of diabetes screening methods or screening for diabetes patients at high risk for other diseases could be steps in the direction of improving HRQOL. Moreover, rural health policy should aim towards new medical interventions and formulating diabetes educational programs for the local population, addressing issues such as obesity, exercise and healthy dieting as preventive measures against the disease.

The SF-36, commonly used in health services research, was chosen as the measuring instrument because it is fairly simple and comprehensible, not time-consuming to answer, and has been previously validated with a representative sample of the Greek population [29]. Furthermore, it is the typical choice when the primary research aim is to measure HRQOL in a specified group rather than to assess the effect of an intervention, in which case a disease-specific instrument should be used as well. A brief comment on the psychometric properties of the instrument in this particular study is that Cronbach's alpha clearly exceeded the recommended 0.7 minimum in all subscales, providing evidence of internal consistency and that each subscale is measuring a distinct concept. Construct validity is supported by the fact that older age, sex (female), diabetic complications, non-diabetic comorbidity and longer duration of diagnosed diabetes all generated impaired SF-36 scores, as was initially expected, and these particular hypotheses have been previously used as validity criteria of the instrument [18].

Our results generally correspond well to findings from previous studies. For example, duration of diabetes was identified as a significant HRQOL predictor in agreement with other studies [6,47], although it has also been shown that the two are insignificantly related [7,19]. The gender effect is quite pronounced in the present study with a worse impact on HRQOL in women. This is consistent with reported gender differences in HRQOL in the general population [29,30], and in studies involving people with diabetes [6,48]. A noteworthy deviation from previous findings is the marked effect of microvascular complications, in contrast to findings in most other studies, where the macrovascular complications and the non-vascular comorbidity show the greatest impact on HRQOL [42]. Although significant diabetes predictors have been identified in this and other studies, the associations do not reveal the underlying mechanism, implying significant space for future research.

This last point highlights a potential limitation to this study. Specifically, concomitant chronic diseases and diabetic complications were self-reported, making it unfeasible to rank these data according to severity, and this could have affected the results in the analyses, since light and severe conditions were grouped together, hence diminishing discriminative ability [42]. This is most likely the case with comorbidity from which 95% of the sample suffers. Furthermore, as studies are comparing diabetes samples with samples from the general population [49,50], it would be interesting to compare HRQOL in this patient group, with an age- and gender-matched group from the Greek rural population, in order to assess the specific impact of the disease. A large-scale population study was recently conducted in Greece, in which HRQOL and other health-related variables were recorded and could be used for forming well-matched general population and disease subgroups for future comparisons.

Finally, one conceptualisation of HRQOL particular to this category of diabetes is disease burden, including patient distress due to diabetes symptoms, complications, or treatment [51]. The use of diabetes-specific HRQOL scales to assess troublesome symptoms and experiences has been recommended for better sensitivity to burden than the SF-36. Advancing age and other health problems may influence health perceptions more than diabetes and undermine reductions in diabetes treatment burden, diabetes related symptoms or disease consequences.

## Conclusion

To conclude, this study has shown Type II DM negatively affects HRQOL in this Greek rural population, particularly in relation to their physical functioning, and that common sociodemographic and clinical indicators such as diabetic complications and non-diabetic comorbidity are important in targeting those at high risk of developing the disease. However, comparison to a control group is required to assess the magnitude of this effect. As the prevalence of DM increases, the disease places more demands on medical care and expenditure. Programs addressing the physical and mental needs of the population are required, especially older people with chronic diseases. The prevention of diabetes, or at least delaying its complications, should become a health priority addressed through education programs delivered to urban and rural citizens.

## Competing interests

The author(s) declare that they have no competing interests.

## Authors' contributions

AP was responsible for designing the study, conducting the literature review and acquiring the data. NK was responsible for analyzing and interpreting the data and drafting the manuscript. AF assisted in interpreting the results and revising the manuscript for intellectual content. EI performed the statistical analysis. DN was responsible for conception of the study. All authors have read and approved the final manuscript.

## Pre-publication history

The pre-publication history for this paper can be accessed here:


